# Measurement of Tetrahydrobiopterin in Animal Tissue Samples by HPLC with Electrochemical Detection—Protocol Optimization and Pitfalls

**DOI:** 10.3390/antiox11061182

**Published:** 2022-06-16

**Authors:** Ksenija Vujacic-Mirski, Matthias Oelze, Ivana Kuntic, Marin Kuntic, Sanela Kalinovic, Huige Li, Jacek Zielonka, Thomas Münzel, Andreas Daiber

**Affiliations:** 1 Department of Cardiology 1–Molecular Cardiology, University Medical Center of the Johannes Gutenberg University Mainz, 55131 Mainz, Germany; ksenija.vujacic.mirski@gmail.com (K.V.-M.); matthias.oelze@unimedizin-mainz.de (M.O.); ivana45@gmail.com (I.K.); marin.kuntic93@gmail.com (M.K.); sanelakalinovic@gmail.com (S.K.); tmuenzel@uni-mainz.de (T.M.); 2 Department of Cardiothoracic and Vascular Surgery, University Medical Center of the Johannes Gutenberg University Mainz, Langenbeckstr. 1, 55131 Mainz, Germany; 3 Department of Pharmacology, University Medical Center of the Johannes Gutenberg University Mainz, Langenbeckstr. 1, 55131 Mainz, Germany; huigeli@uni-mainz.de; 4 Department of Biophysics, Medical College of Wisconsin, Milwaukee, WI 53226, USA; jzielonk@mcw.edu; 5 German Center for Cardiovascular Research (DZHK), Partnersite Rhine-Main, 55131 Mainz, Germany

**Keywords:** tetrahydrobiopterin, HPLC with electrochemical detection, oxidative stress, sepsis

## Abstract

Tetrahydrobiopterin (BH4) is an essential cofactor of all nitric oxide synthase isoforms, thus determination of BH4 levels can provide important mechanistic insight into diseases. We established a protocol for high-performance liquid chromatography/electrochemical detection (HPLC/ECD)-based determination of BH4 in tissue samples. We first determined the optimal storage and work-up conditions for authentic BH4 and its oxidation product dihydrobiopterin (BH2) under various conditions (pH, temperature, presence of antioxidants, metal chelators, and storage time). We then applied optimized protocols for detection of BH4 in tissues of septic (induced by lipopolysaccharide [LPS]) rats. BH4 standards in HCl are stabilized by addition of 1,4-dithioerythritol (DTE) and diethylenetriaminepentaacetic acid (DTPA), while HCl was sufficient for BH2 standard stabilization. Overnight storage of BH4 standard solutions at room temperature in HCl without antioxidants caused complete loss of BH4 and the formation of BH2. We further optimized the protocol to separate ascorbate and the BH4 tissue sample and found a significant increase in BH4 in the heart and kidney as well as higher BH4 levels by trend in the brain of septic rats compared to control rats. These findings correspond to reports on augmented nitric oxide and BH4 levels in both animals and patients with septic shock.

## 1. Introduction

Tetrahydrobiopterin (BH_4_, sapropterin) is a cofactor of the three aromatic amino acid hydroxylase enzymes and nitric oxide synthases (NOS). The hydroxylase enzymes convert aromatic amino acids such as phenylalanine, tyrosine, and tryptophan to precursors of dopamine and serotonin, which act as major monoamine neurotransmitters [1]. The NOS enzyme family produces nitric oxide (NO), a mediator of endothelium-dependent vasodilation, inhibitor of platelet aggregation and regulator of smooth muscle tone, cell growth and differentiation [2]. BH_4_ is sold under the brand names Kuvan and Biopten for supplementation in BH_4_ deficiency caused by genetic inactivity of GTP-cyclohydrolase-I (GTPCH-I) or 6-pyruvoyltetrahydropterin synthase (PTPS), both enzymes involved in BH_4_ synthesis as encountered in phenylketonuria [3].

BH_4_ is an important regulator of cardiovascular development and homeostasis [4]. BH_4_ deficiency has been reported in almost all cardiovascular diseases and has been suggested to play an important role in the development of endothelial dysfunction and progression of atherosclerosis [4,5,6,7,8]. Oxidative loss of BH_4_ triggers the uncoupling of endothelial NOS (eNOS) in the conditions of oxidative stress that are present in hypertension, diabetes, hypercholesterolemia and atherosclerosis [2,9,10,11,12]. Oxidation of BH_4_, e.g., in the presence of peroxynitrite, leads to formation of 7,8-dihydrobiopterin (BH_2_) [13,14], which can be further oxidized to biopterin and other products. Since BH_4_ bioavailability is tightly linked to regulation of eNOS activity, the levels of BH_4_ have a strong impact on vascular function, cardiovascular health and the adhesion/infiltration of immune cells [15,16,17,18]. On the other hand, induction of BH_4_ synthesis is a hallmark of severe inflammatory conditions, as observed in the setting of endotoxemia (e.g., by lipopolysaccharide [LPS]) and septic shock [19,20,21]. A similar increase in BH_4_ synthesis and nitric oxide formation was observed in a more moderate model of cardiovascular inflammation, mice with angiotensin-II-induced arterial hypertension [18]. Generally, inflammation is associated with upregulation of GTPCH-I, providing higher BH_4_ concentrations for inducible nitric oxide synthase (iNOS) [18,22].

In light of the aforementioned central role of BH_4_ in endothelial dysfunction as well as inflammation-associated complications, the exact determination of BH_4_ is of great importance. BH_4_ can be detected by indirect methods upon the differential oxidation of BH_4_ and BH_2_ to fluorescent biopterin (iodine method) as well as by direct methods using post-column electrochemical oxidation or MS/MS-based detection. Indirect methods rely on liquid chromatography coupled to UV/Vis or fluorescence detection of BH_4_ oxidation products [23]. Here, we used the HPLC/ECD method for the reliable (and direct) detection of BH_4_ and BH_2_ concentrations in standards as well as BH_4_ levels in samples of control and septic rats. We also used the HPLC/ECD method for evaluation of the optimal storage and work-up conditions for BH_4_- and BH_2_-containing samples.

## 2. Materials and Methods

### 2.1. Chemicals

Tetrahydro-L-biopterin (BH_4_) hydrochloride and 7,8-dihydro-L-biopterin (BH_2_) hydrochloride were obtained from Cayman Chemical Company (Ann Arbor, MI, USA) (CAS: 69056-38-8). Hydrogen peroxide 30% solution (CAS: 7722-84-1), 1,4-dithioerythritol ≥ 99.0% (DTE) (CAS: 6892-68-8), diethylenetriaminepentaacetic acid 98% (DTPA) (CAS: 67-43-6), LPS from Escherichia coli (L-4005) and Salmonella typhosa (L6386), potassium dihydrogen phosphate (# P018.2) and phosphorus acid (# 9079.1) were obtained from Sigma, Merck KGaA, Darmstadt, Germany.

### 2.2. Stability of BH_4_ and BH_2_ Standard Stock Solutions under Different Storage Conditions

BH_4_ and BH_2_ standard stock solutions of 1 mM were prepared in 100 µM HCl with or without 1 mM DTE and 1 mM DTPA, which were then divided and further diluted to 100 µM for overnight storage and up to 2 weeks at different temperatures (4 °C, −20 °C and −80 °C). Some standard stock solutions were incubated with 1 mM hydrogen peroxide overnight at 4 °C in order to mimic the autoxidation of BH_4_ at ambient oxygen concentrations. After applying the different storage conditions and times, the samples were subjected to HPLC/ECD analysis.

### 2.3. Animals

All animals were treated in accordance with the Guide for the Care and Use of Laboratory Animals as adopted by the U.S. National Institutes of Health and approval was granted by the Ethics Committee of the University Hospital Mainz and the Landesuntersuchungsamt Rheinland-Pfalz (Koblenz, Germany; permit number: 23 177-07/G 18-1-001). Male Wistar rats (6 weeks old, 300 g, Charles River Laboratories, Sulzfeld, Germany) were used for the study and all efforts were made to minimize suffering. Diabetes was induced with a single injection of streptozotocin (60 mg/kg) in the dorsal vein of the penis with incubation of animals for 6 weeks [24,25], while sepsis was induced by intraperitoneal injection of LPS (from Escherichia coli and Salmonella typhimurium in ratio 3:1, 10 mg/kg) 12 h before sacrifice [26,27]. Animals were killed under isoflurane anesthesia by transection of the diaphragm and exsanguination. Aorta, heart, brain, liver and kidney tissues were harvested for further analysis.

### 2.4. HPLC/ECD

For the electrochemical detection of BH_4_ and BH_2_, an UltiMate 3000 system with Dionex™ CoulArray™ (Coulometric Array Detector) (Thermo Fisher Scientific GmbH, Dreieich, Germany) was used—a high-quality instrument designed for high sensitivity detection of electroactive molecules. The system is controlled by two different software programs: Chromeleon Chromatography Management System (Chromeleon) software and CoulArray software. Chromeleon software controls the injection volume of sample, flow, mobile phase gradient and functions of the autosampler. The CoulArray software controls the temperature of the column and the two coulometric multi-channel cells, as well as the setup of different electrochemical potentials. A coulometric cell with a large surface area consisting of porous graphite electrode material allows complete oxidation (or reduction) of the electrochemically active molecules, minimizing the noise and providing enhanced sensitivity. Each sample run ends with a short cleaning procedure of the electrochemical cells (setting all cells to 800 mV).

For detection of BH_4_ (protocol A), potentials of 0, +150, +280, +365, +600 mV (vs. palladium reference electrode) were used. The BH_4_ peak yielded the most pronounced signal at 280 mV, whereas the BH_2_ peak showed the highest signal at 600 mV. For separation, an analytical column Synergi Polar-RP (Phenomenex, Torrance, CA, USA, 4 µm, 80 Å, 4.6 × 250.0 mm) was used at 37 °C column temperature (4 °C autosampler rack temperature). A sample volume of 20 µL was injected. The mobile phase consisted of 50 mM potassium phosphate, pH 2.6, with 0.1 mM DTE and 0.1 mM DTPA using an isocratic protocol, with the flow rate set at 0.7 mL/min. External and internal BH_4_/BH_2_ standards (by “spiking”) were used for peak identification and quantification. Typical retention times were 5.3 min for BH_4_ and 9.2 min for BH_2_. Protocol A was adapted from previous work [28,29]. Unfortunately, ascorbate co-eluted with BH_4_ in this HPLC method.

For the analyses of tissue samples, we established a second protocol, protocol B, with a clear separation of BH_4_ and ascorbate peaks. 50 mM potassium phosphate was set to pH 4.5 (based on other reports using higher pH of the mobile phase [30,31]), without DTE and DTPA using isocratic protocol with flow rate set at 1 mL/min while cell potentials were set to 0 and +150 mV. This condition provided better separation of interfering peeks in the animal tissue with BH_4_ eluting at 4.6 min with the most pronounced signal observed at 0 mV. All experimental parameters for optimized HPLC/ECD-based detection of BH_4_ and BH_2_ used in both protocols are shown in Table 1.

### 2.5. Animal Tissue Preparation

**Figure 1:**
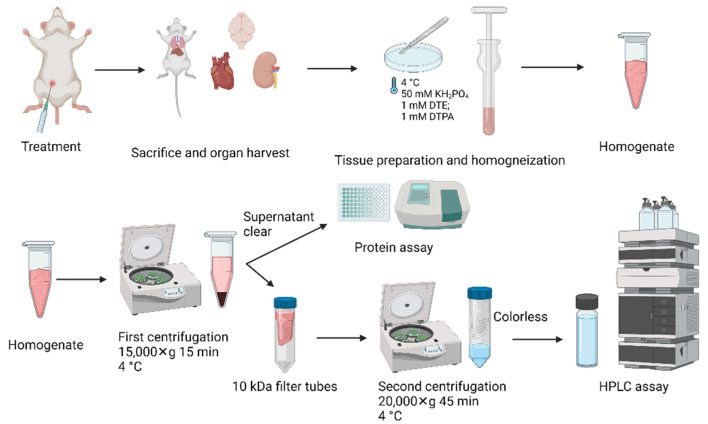
Scheme with work-up conditions for harvesting and processing of animal tissues. Created with Biorender.com (last accessed 14 June 2022).

After sacrifice, rat tissues (aorta, heart, brain, kidney and liver) were immediately washed and briefly stored in ice-cold homogenization solution consisting of 50 mM potassium phosphate buffer pH 2.6 with 1 mM DTE and 1 mM DTPA. Tissue was cleaned from extra fat (aorta was cleaned in homogenization buffer under the microscope on ice). The wet weight of all the tissues was measured before homogenization. Roughly, half of a heart (~700 mg), half of a kidney (~1000 mg), similar piece of a liver or a brain (~1000 mg) and the whole aorta (~150 mg) were used for BH_4_ analysis. Samples were cut into small pieces, followed by glass–glass homogenization at 4 °C upon addition of 1 mL of homogenization buffer for heart, brain, kidney and liver tissue and 700 µL for aorta. The samples were centrifuged for 15 min at 15,000× *g*. Supernatant (slightly colored) was taken and centrifuged again through 10 kDa cut-off filters for 45 min 20,000× *g*. After centrifugation the completely colorless supernatant was loaded to the autosampler. All centrifugation steps and the autosampler were kept at 4 °C. Supernatant of the first centrifugation step was used for quantification of protein content by Lowry method. All work-up steps and conditions are summarized in Figure 1.

### 2.6. Statistical Analysis

Results are expressed as mean ± SD. Unpaired t-test was used for all BH_4_ data obtained in tissues; all showed normal distribution, but some failed equal variance where Welch correction was used. Non-parametric test was used for all BH_4_ data obtained in standard stock solutions (GraphPad Prism 9.0.1 for Windows.). *p*-Values < 0.05 were considered statistically significant.

## 3. Results

### 3.1. Standard Curve

**Figure 2:**
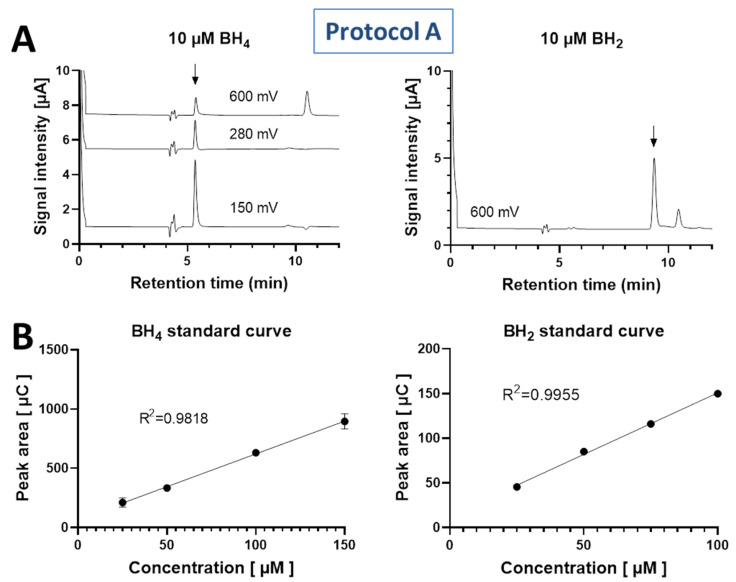
(**A**) Chromatograms of 100 µM BH_4_ and BH_2_ standards represented at oxidation potentials of 150, 280 and 600 mV for BH_4_ (retention time 5.4 min) and at the oxidation potential of 600 mV for BH_2_ (retention time 9.4 min). (**B**) Standard curves for BH_4_ and BH_2_ solutions, representing a linear range (25–150 and 25–100 µM concentration) with a linear regression correlation coefficient R^2^ of >0.98 for both standards. Data are mean ± SD of n = 4 independent measurements.

BH_4_ was detected at the lower potentials of 150 mV and 280 mV and showed a peak at a retention time of 5.35 min, whereas BH_2_ was detected at the high potential of 600 mV and showed a peak at a retention time of 9.38 min (Figure 2A). Standard curves for both BH_4_ and BH_2_ were linear in the concentration range of 25 to 150 or 100 µM with R^2^ values of >0.98 (Figure 2B).

### 3.2. Stability of Standards during Storage

**Figure 3:**
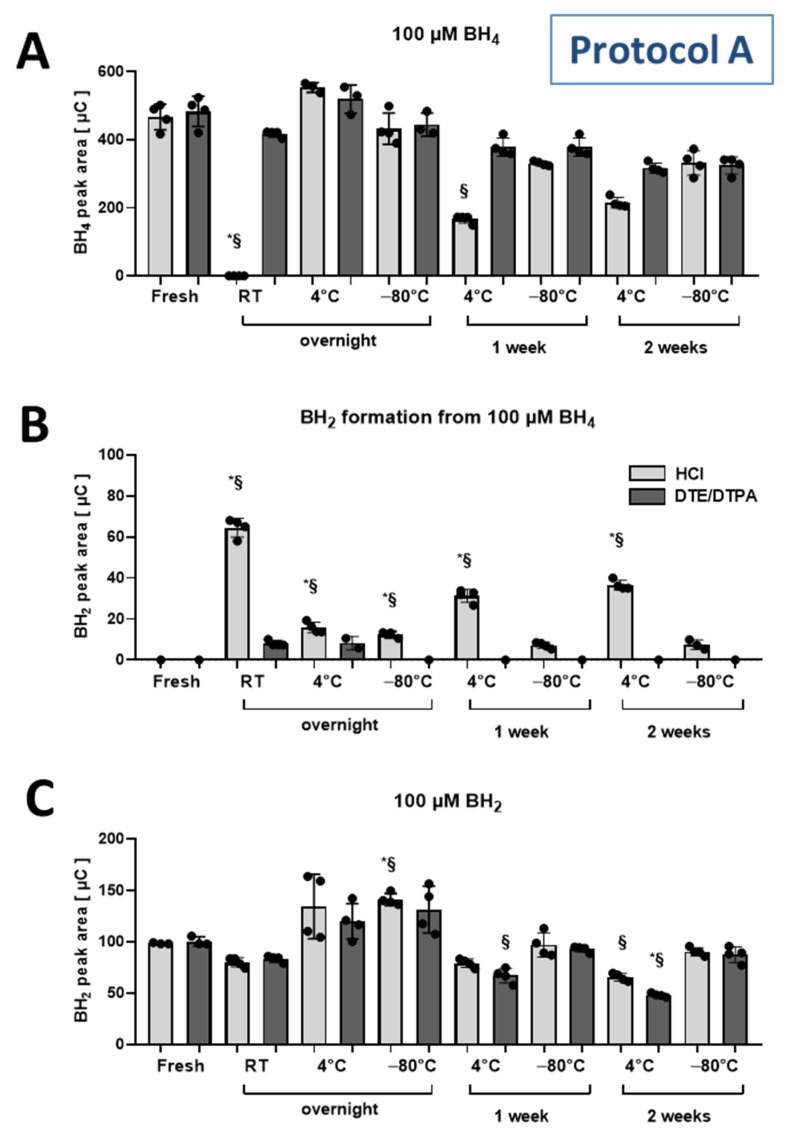
(**A**) Detected BH_4_ peak areas in solutions of BH_4_ (100 µM) standards (quantified as the sum of 150 mV and 280 mV oxidation potentials, eluted at 5.4 min) upon storage under indicated conditions and time. (**B**) Peak areas of BH_2_ detected (quantified at 600 mV oxidation potential, retention time 9.4 min) in the same samples. (**C**) Detected BH_2_ peak areas in solutions of BH_2_ (100 µM) standards (quantified at 600 mV oxidation potential, eluted at 9.4 min) upon storage under different conditions. Solvent conditions were either hydrochloric acid (HCl, 100 µM) or HCl (100 µM) with dithioerythritol (DTE, 1 mM) and diethylenetriaminepentaacetic acid (DTPA, 1 mM). Data are mean ± SD of the number of indicated independent measurements. Note: * means significantly different to fresh standard in HCl; § means significantly different to fresh standard in HCl with DTE/DTPA.

Standards were prepared in 100 µM HCl (plus acidity from BH_4_ and BH_2_ hydrochlorides used) with or without 1 mM DTE and 1 mM DTPA. These standards were either measured directly after preparation or upon storage at room temperature, 4 °C or −80 °C overnight, for 1 week or for 2 weeks. BH_4_ was completely decomposed and BH_2_ was formed upon overnight storage at room temperature when only dissolved in HCl solution. Even when stored at 4 °C, BH_4_ showed appreciable degradation after one or 2 weeks of storage when only dissolved in HCl. The presence of DTE and DTPA stabilized BH4 standards at all temperatures. Only at longer storage times (weeks) there was up to 20% unspecific loss of BH_4_, even at −80 °C. BH_2_ standards were stable under all conditions in HCl solution. Graphs for BH_4_ and BH_2_ signal response after storage under different conditions are shown in Figure 3.

### 3.3. Oxidation of Standards

**Figure 4:**
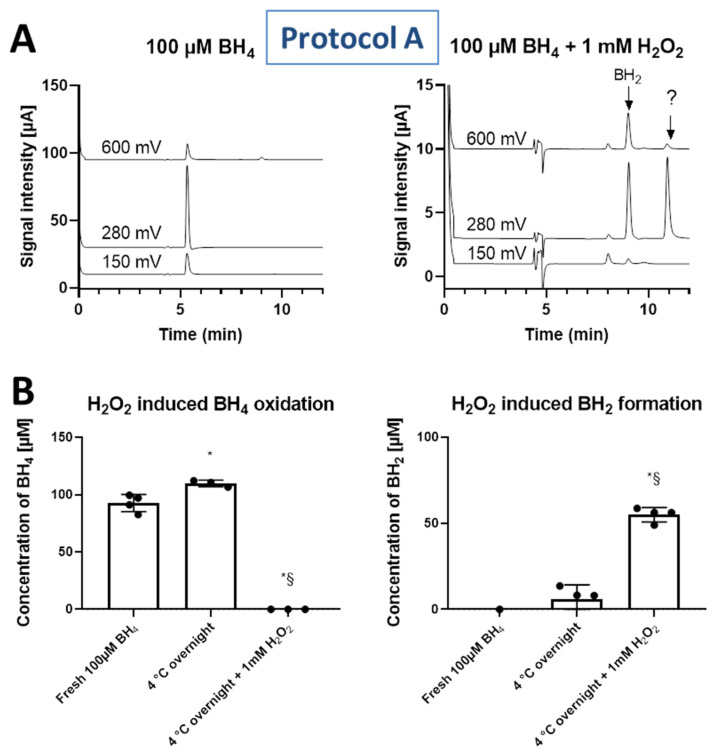
(**A**) Representative chromatograms showing the changes in peak intensities due to BH_4_, BH_2_ and unidentified product (marked with “?” symbol, retention time approximately 11 min) in BH_4_ (100 µM) solutions incubated in the presence or absence of H_2_O_2_ (1 mM). (**B**) Concentrations of BH_4_ standard and formed BH_2_ product (quantified as the sum of 150 mV and 280 mV oxidation potentials for BH_4_, and only 600 mV for BH_2_) were either measured in freshly prepared samples in 100 µM HCl or after incubation overnight at 4 °C or after incubation overnight at 4 °C in the presence of 1 mM H_2_O_2_ in 0.1 mM HCl. Data are mean ± SD of the number of indicated independent measurements. Note: * means significantly different to fresh BH_4_; § means significantly different to overnight BH_4_.

Oxidation of 100 µM BH_4_ standard (prepared in 100 µM HCl solution) by 1 mM H_2_O_2_ was performed by overnight incubation at 4 °C. BH_4_ was completely decomposed and there was notable formation of BH_2_ as well as another, unidentified degradation product with the retention time of approximately 11 min (Figure 4).

### 3.4. Animal Tissue

**Figure 5:**
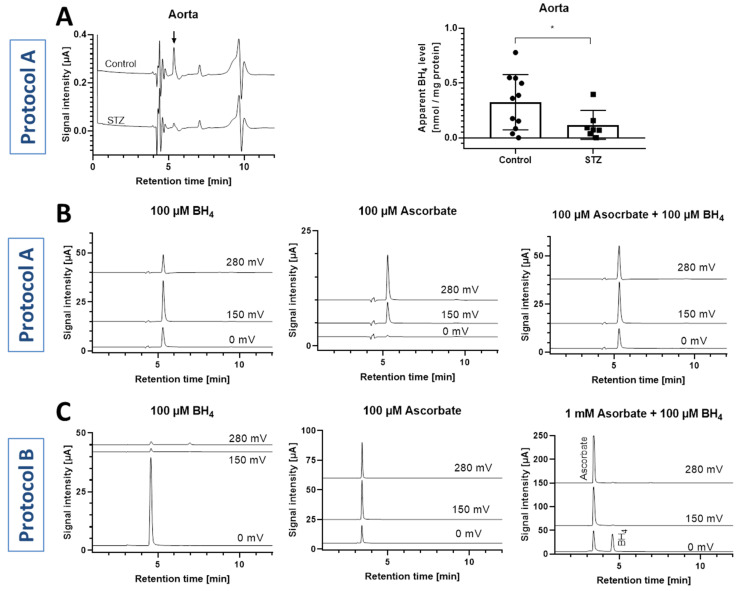
(**A**) Representative chromatograms (detected at 280 mV oxidation potential) and determined apparent BH_4_ concentrations for aorta of control and diabetic (STZ) rats. Peak areas detected at the retention time of 5.4 min were converted to a BH_4_ concentration using the BH_4_ standard curve and was finally normalized to mg protein as estimated by Lowry method in the tissue homogenate supernatant. Data are mean ± SD of the number of indicated independent measurements. Note: * means significantly different to control rats. (**B**) Representative chromatograms showing co-elution of BH_4_ and ascorbate standards using the initial HPLC method (protocol A). (**C**) Representative chromatograms show clear separation of BH_4_ and ascorbate standards using the optimized HPLC method (protocol B).

Using the first HPLC protocol A buffer and settings (50 mM potassium phosphate, pH 2.6, with 0.1 mM DTE and 0.1 mM DTPA), a signal was detected in all animal tissues (aorta, heart, kidney, liver and brain) that were eluted with the retention time of BH_4_, which was only significantly reduced in aortic tissue of diabetic (STZ) rats (Figure 5A). BH_2_ could not be detected in any of these tissues. We were, however, surprised by the magnitudes higher concentration of BH_4_ in all of these tissues as compared to previously published data and realized that ascorbate often co-elutes with BH_4_ and shows comparable electrochemical properties, as stressed previously [32]. Further analysis showed that, indeed, BH_4_ and ascorbate standards co-eluted at the same time (Figure 5B). We therefore disregarded all data obtained from the tissues of the diabetic rats.

**Figure 6:**
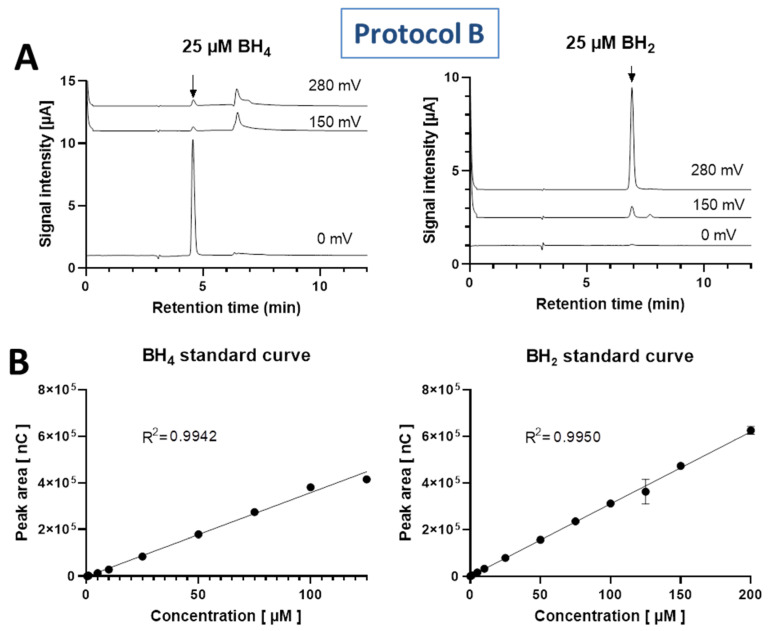
(**A**) Chromatograms of 25 µM BH_4_ and BH_2_ standards represented at oxidation potentials of 0, 150 and 280 for BH_4_ (retention time 4.56 min) and at the oxidation potential of 280 mV for BH_2_ (retention time 6.92 min). (**B**) Standard curves for BH_4_ and BH_2_ solutions, representing a linear range (BH_4_: 0.3–125 µM, 13 concentration values; BH_2_: 0.1–200 µM, 15 concentration values) with a linear regression correlation coefficient R^2^ of 0.99 for both standards. Data are mean ± SD of n = 3 independent measurements per data point.

After optimization of the HPLC method by increasing the pH of the mobile phase (second protocol B: 50 mM potassium phosphate, pH 4.5), we were able to separate the BH_4_ signal from the interfering ascorbate peak (Figure 5C). BH_4_ was detected at the lower potential of 0 mV and showed a peak at a retention time of 4.56 min, whereas BH_2_ was detected at the higher potential of 280 mV and showed a peak at a retention time of 6.92 min (Figure 6A). Standard curves for both BH_4_ and BH_2_ were linear in the concentration range of 0.3/0.1 to 125/200 µM with R^2^ values of >0.99 (Figure 6B).

**Figure 7:**
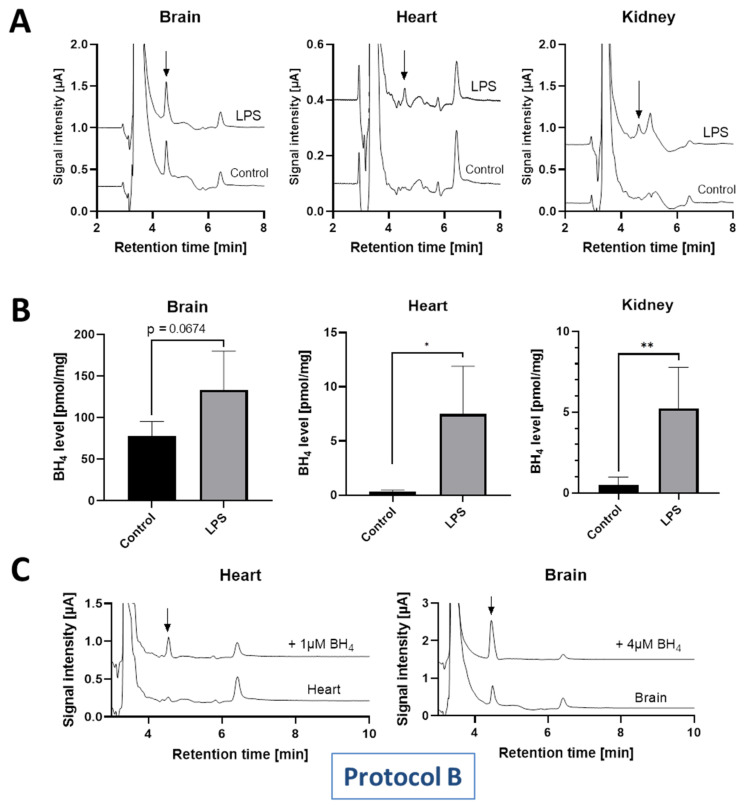
(**A**) Representative chromatograms (measured at 0 mV potential) of BH_4_ in the brain, heart and kidney of control and LPS-treated septic rats. (**B**) Concentration levels in these tissues as calculated from BH_4_ standard curve. Data are mean ± SD of the number of indicated independent measurements. Note: * means significantly different to control rats. *, *p*-value is < 0.05; **, *p*-value is < 0.01 to indicate significance against the control. (**C**) Representative chromatograms (measured at 0 mV potential) of BH_4_ in the heart and brain of control rats with and without spiking with authentic BH_4_ standard.

Unfortunately, the BH_4_ signals in the tissues of control rats were then too low to observe a significant decrease in the tissues of diabetic rats (not shown). We therefore chose to monitor the induction of BH_4_ under inflammatory conditions instead, using LPS-induced endotoxemia. We successfully detected BH_4_ in the brain, heart and kidney of control and LPS treated rats in the concentration range more similar to previously published data (Table 2). LPS-induced sepsis is known to increase nitric oxide and BH_4_ levels via inflammatory pathways via induction of iNOS [19,20,21,22]. Chromatogram peaks of BH_4_ in the brains were detectable in control and LPS-treated rats (Figure 7A). Heart and kidney levels of BH_4_ showed a significant increase in LPS-treated animals as compared to the control, while brain BH_4_ concentrations were increased in LPS animals, at least by trend (*p*-value 0.067) (Figure 7B). In order to prove the authenticity of the potential BH_4_ signal in the heart and brain, the samples were spiked with BH_4_ standard (Figure 7C).

## 4. Discussion

With the present studies, we systematically investigated the effect of storage conditions and time on stability of BH_4_ and BH_2_ and established a protocol for their quantification in tissues by using HPLC with electrochemical (coulometric) detection (HPLC/ECD). We showed here that storage conditions play an important role in the stability of BH_4_ standards and probably also in tissue samples containing BH_4_. Whereas low temperatures in general improve the stability of BH_4_ during storage, the addition of DTE and DTPA represents the major determinant for prevention of BH_4_ degradation during storage. BH_2_ is much less susceptible to degradation during storage with respect to temperature conditions and the addition of an antioxidant or a metal chelator, and acidification of the solutions seemed to be sufficient to stabilize BH_2_. With the HPLC/ECD protocol A for BH_4_ and BH_2_ quantification that was optimized from a published method [28,29], we were able to determine the optimal storage conditions. We applied the optimized storage conditions to tissue samples from control and diabetic rats and detected a peak with the retention time of BH_4_. Quantitative analyses, however, suggested that BH_4_ levels are significantly higher than previously reported (Table 2). Additional experiments revealed the co-elution of ascorbate and BH_4_ under the chosen chromatographic conditions, a problem previously reported for tissue BH_4_ analyses [32]. For selective detection of BH_4_, we further modified the chromatographic elution conditions and obtained satisfactory separation of BH_4_ and ascorbate. Using this improved method, we were able to selectively measure BH_4_ in the tissue samples and observed an increase of BH_4_ levels in different tissues of septic (LPS-treated) rats.

Different electrochemical methods have been developed for sensitive pteridines detection taking advantage of their electrochemical properties. Volt/amperometry has been used extensively for qualitative and quantitative determination of pteridines. The first HPLC methods used for the estimation of BH_2_ and BH_4_ levels used iodine in acidic or alkaline solution to differentially oxidize BH_2_ and BH_4_ to biopterin and calculate the individual concentrations of BH_2_/BH_4_ from the difference of the acidic and alkaline determination [43]. Oxidation can also be achieved by manganese dioxide [44,45]. These methods did not include electrochemical detection, but rather they were using oxidizing conditions to create products such as biopterin which could be detected and identified by chromatography coupled with optical (UV or fluorescence) detection. Because those assays are involved in the conversion of BH_4_ and BH_2_ into biopterin, these can be regarded as indirect methods. Direct methods are the means of choice for the most accurate and precise detection of cellular metabolites and the state-of-the-art methods for BH_4_ and BH_2_ detection that are based on the use of high performance liquid chromatography (HPLC) coupled with either electrochemical detection (HPLC/ECD) [28,30,33,38] or mass spectrometric detection (LC-MS/MS) [41]. By using HPLC coupled with both electrochemical and fluorescence detectors, even simultaneous quantification of BH_4_, BH_2_ and biopterin can be achieved [46]. Unlike the use of fluorescent methodology only, the multi-electrode coulometric detection enables the direct measurement of both BH_4_ and/or BH_2_ within the same analysis of cell extract at applied potentials ≥280 mV for BH_2_ and ≤150 mV for BH_4_ [47]. In addition to using only positive potentials, there are methods, which rely on reversibility of the oxidized product by keeping one electrode at negative potential [48]. In the present study, using the protocol B optimized for tissue samples, BH_4_ was determined using the first electrochemical channel, set at 0 mV vs. the palladium reference electrode.

We used DTE and DTPA agents to prevent autoxidation of BH_4_ and BH_2_. These agents were added to standards, samples and to the mobile phase. In addition to autoxidation in biological samples, reducing agents such as DTE can prevent iron-catalyzed oxidative breakdown of BH_4_ [49]. Iron proteins are abundant in cells, leading to continuous oxidation of BH_4_ [50]. DTPA is specially effective for preventing redox reactions by metal ions, such as Fe(II)/(III), Mn(II)/(IV) and Cu(I)/(II), that induce oxidative damage by superoxide and hydrogen peroxide [51]. In line with these considerations, the addition of DTE/DTPA largely prevented oxidative degradation of BH_4_ standards, even at higher temperature storage conditions: overnight at room temperature or up to two weeks at 4 °C.

LC-MS/MS is mainly used to separate, detect, identify and quantify biomolecules in samples in the presence of complex chemical mixtures. Nevertheless, the HPLC-ECD method performed well in terms of various validation parameters for detection of artesunate and dihydroartemisinin when compared head to head with LC-MS/MS analysis [52]. Additionally, head-to-head comparison of HPLC/ECD-based quantification of BH_4_ or HPLC/fluorescence detection of neopterin in human plasma of individuals with genetic defects in BH_4_ and pterin synthesis revealed a small variation from the respective values determined by LC-MS/MS analysis [53]. Whereas the LC-MS/MS method may require more laborious sample preparation, HPLC-ECD requires clear separation of compounds in order to clearly confirm their identity, e.g., by “spiking” with authentic standard. Both techniques have problems with the identification of structural isomers that show similar retention times. LC-MS/MS is a relatively expensive method with respect to the instrument itself as well as maintenance costs. Another major disadvantage of LC-MS/MS is that it works most accurately with volatile buffers. Buffer salts represent a major problem for most LC-MS/MS systems. Direct determination of BH_4_ in biological samples by optical methods is difficult due to low sensitivity and a lack of fluorescence of BH_4_ and BH_2_. Calibration curves for BH_4_ and BH_2_ analysis can hardly be found in published work, especially for HPLC with fluorescence or electrochemical detection. Rather old work reported limits of detection for BH_4_ of 1.24 pmol in 200 µL, corresponding to 6.2 nmol/L when using the differential oxidation and HPLC with fluorescence detection method [43]. For HPLC/ECD, old studies reported limits of detection for BH_4_ of 0.94 pmol in urine [54] and 0.19 pmol in liver and brain [55]. The combination of electrochemical oxidation and subsequent fluorescence detection allowed measurement with limits of detection for dihydroxanthopterin, 6-biopterin, pterin, BH_2_ and BH_4_ of 120, 80, 160, 100 and 60 fmol, respectively [56]. More recent work using the LC-MS/MS method reported a limit of quantitation for BH_4_ of 10 ng/mL (41.5 nmol/L) in mouse plasma after oral versus intravenous administration of BH_4_, also allowing kinetic monitoring of the uptake, body distribution and half-life of BH_4_ [39]. Inaccuracy and imprecision were ≤15% and recovery of BH_4_ was 80% when samples were stored for 24 h in the presence of DTE [39]. Alternatively, BH_4_ detection upon derivatization with benzoyl chloride coupled with liquid chromatography–tandem mass spectrometry analysis provided a limit of quantification of 0.02 ng/mL (83 pmol/L) in human plasma, and BH_4_ levels inversely correlated with age [40]. Other LC/tandem mass spectrometry methods established a limit of quantification of 1 nmol/L for BH_4_ and BH_2_ and 2.5 nmol/L for biopterin in cultured endothelial cells [57] and of 1 ng/mL (4.2 nmol/L) for BH_4_ in human plasma [58]. More discussion of the different analytical methods for BH4 quantification can be found in reference [34].

Oxidation reactions of tetrahydropterins proceed via complex mechanisms to initially give 6H-7,8-dihydro derivatives, which are stabilized by prototrophy to the 7,8-dihydro isomers [59]. Under cellular conditions, BH_4_ is oxidized to 4a-tetrahydrobiopterin, which is afterwards recycled by 4a-hydroxytetrahydrobiopterin dehydratase or undergoes spontaneous dehydration to 6,7-dihydrobiopterin, and 6,7-BH_2_ can be reduced back to BH_4_ by 6,7-dihydropteridine reductase; however, in the absence of sufficient 6,7-dihydropteridine reductase activity, the quinoid 6,7-BH_2_ rearranges non-enzymatically into the more stable 7,8-BH_2_ (BH_2_) [60]. In the system of H_2_O_2_-induced oxidation of BH_4_, we observed the formation of BH_2_. BH_4_ synthesis and restoration is mainly based on two important enzymes: GTPCH-I, already mentioned above, and dihydrofolate reductase (DHFR), an enzyme which is responsible for recycling of oxidized BH_2_ back to BH_4_ [61,62]. Therefore, they also represent pharmacological targets for the correction of endothelial dysfunction and the prevention of the progression of cardiovascular disease [63,64], as also demonstrated by the prevention of vascular complications in diabetic or hypertensive animals by endothelium-specific overexpression of GTPCH-I [11,65]. As suggested previously, a combination of antioxidant therapy and agents capable of increasing intracellular BH_4_ levels may be required in order to successfully treat cardiovascular diseases, so precursors of BH_4_, such as sepiapterin, along with antioxidants, may represent the most promising strategy [64,66].

The initial attempts to measure BH_4_ levels in diabetic rats and to confirm the reported BH_4_ decrease failed because of co-elution of BH_4_ with ascorbate, a known problem, especially since tissues such as the brain are very rich in ascorbate [32,67]. Upon further modification of the HPLC method, we were able to fully separate BH_4_ and ascorbate peaks (protocol B). The main finding of our work is the straightforward detection of BH_4_ in tissue samples and increased BH_4_ levels in different organs of septic animals. The BH_4_ peak identity was confirmed by ”spiking“ the samples with small amounts of authentic BH_4_ standard and observing the extent of the increase in the intensity of the peak of interest. In the literature, a large concentration range of BH_4_ tissue levels were reported, depending on the species, the organ, the health state of the animals and the measurement protocols (see Table 2, more ECD/HPLC examples in [50,68,69,70]). The BH_4_ concentrations determined in this report are at the lower range of the published values. Whereas, in human endothelial cells (HAECs and HUVECs), concentrations of BH_4_ of approximately 0.3 pmol/mg protein were found using an HPLC/ECD method, the level in bovine endothelial cells (BAECs) was 35 pmol/mg protein, and in mouse endothelial cells (sEnd.1) it was 280 pmol/mg protein [50].

The BH_2_ content in these cells ranged from 2.6 to 120 pmol/mg protein. The strong link between BH_4_ levels and inflammation (necessary to provide enough BH_4_ for iNOS activity) was evident from previously reported HPLC/ECD data in septic mice, where plasma BH_4_ levels increased from 40 to 150 nM within 6 h after induction of sepsis with a subsequent decrease to 70 nM within 24 h after induction of sepsis [22]. In accordance with the changes shown in plasma BH_4_ concentration, these authors showed that BH_4_ levels increased from 5 to 8 pmol/mg protein in the heart 6 h after sepsis and were decreased to 3 pmol/mg protein 24 h after starting the experiment. No data on BH_2_ levels were, however, reported in that study. In another study, the determined concentration of BH_4_ in the pancreas was 25 pmol/mg protein (80 pmol/mg after BH_4_ administration) before ischemia and 20 pmol/mg protein (45 pmol/mg after BH_4_ administration) afterwards [71]. The data presented in Table 2 stresses the large variability in BH_4_ tissue levels. Of note, it is striking that only two of the reports using the HPLC/ECD method also provided BH_2_ data for renal tissue that showed at least a 5- or 10-fold increase in diabetic rats and was reduced by ebselen antioxidant therapy to control levels [30,38]. Most published data on tissue BH_2_ levels are based on the HPLC/fluorescence method with differential oxidation of BH_4_/BH_2_, although with only marginal effects of disease or age on BH_2_ level [35,36] and a striking increase in mice overexpressing the GTPCH-I enzyme [37]. It should be also kept in mind that BH_2_ data obtained by using the HPLC/fluorescence method with differential oxidation represent a sum of “BH_2_ plus biopterin” present in the tissue. From these literature data we conclude that BH_2_ levels are rather low and do not change much in response to many disease conditions when analyzed by the HPLC/ECD method.

As for the biological importance of our findings, BH_4_ represents a critical regulator of eNOS activity and thereby of endothelial function, determining the vascular tone and anti-adhesive properties of the endothelial cell layer [64]. The oxidative degradation of BH_4_ as a mediator of eNOS uncoupling is best characterized as a “redox switch” in eNOS activity and supported by studies on hypertension, diabetes, hypercholesterolemia [72] and atherosclerosis [2,9,10,11,12,13]. The depletion of BH_4_ in almost all cardiovascular diseases is also well established and frequently reviewed [4,5,6,7,8]. The most important enzyme for de novo synthesis of BH_4_ is GTPCH-I, which was identified as a central regulator of eNOS activity and endothelial function [15], as is also supported by data on eNOS uncoupling and impaired endothelial function in mice with endothelial-specific genetic deletion of GTPCH-I (Gch1^fl/fl^Tie2cre mice) [16,17]. The ratio of eNOS and GTPCH-I expression levels represents a major determinant of healthy endothelial function, and transgenic overexpression of eNOS without matched elevation of BH_4_ concentrations ultimately leads to uncoupled eNOS enzyme [12].

Peroxynitrite (ONOO^−^), a product of a diffusion-controlled reaction between nitric oxide and superoxide [73,74], not only causes oxidative depletion of BH_4_ but also can induce proteasomal degradation of GTPCH-I by oxidative activation of the 26S proteasome [32,75,76]. In addition to GTPCH-I, DHFR is important for BH_4_ bioavailability as recycling of oxidized BH_2_ back to BH_4_ constitutes the “salvage pathway” [61,62]. The oxidative activation of the 26S proteasome was also shown to be involved in the proteasomal degradation of DHFR, which was inhibited by eNOS-dependent nitric oxide formation and S-nitrosation of DHFR [77]. Accordingly, the BH_4_ system has multiple targets for the prevention of endothelial dysfunction and the mitigation of adverse cardiovascular health effects [63]. In line with the presented data, a combination of antioxidants and BH_4_ therapy is recommended to successfully treat endothelial dysfunction and vascular oxidative stress in the setting of cardiovascular diseases [66]. Acute infusion of high local concentrations of BH_4_ in smokers [78] and diabetic patients [79] improved endothelial-dependent dilation. Importantly, in vitro, NH_4_ scavenged superoxide anion radicals created by the xanthine/xanthine oxidase reaction equipotent to BH_4_ but failed to modify acetylcholine-induced changes in forearm blood flow in chronic smokers in vivo, supporting the concept that, in addition to the reactive oxygen species burden of cigarette smoke, a dysfunctional eNOS due to BH_4_ depletion may contribute, at least in part, to endothelial dysfunction in chronic smokers [78].

Similarly, administration of the BH_4_ precursor folic acid or sepiapterin restored impaired endothelial function in healthy subjects [80,81] as well as in hypertensive and atherosclerotic mice [13,82]. However, despite strong evidence from preclinical and human cohort studies on a therapeutic benefit of eNOS “recoupling” by the administration of BH_4_ [78] or its precursors (e.g., sepiapterin or folate) [83], this concept was, until now, not translated to clinical therapy, except for the treatment of phenylketonuria [3]. Therefore, the clinical data are so far inconclusive and warrant larger clinical trials on the cardio/cerebrovascular effects of BH_4_-related drugs. Based on the observed instability of BH_4_ under oxidative environment, it is also possible that the use of BH_4_ precursors may be a more efficient strategy to increase its intracellular levels. Therefore, a sensitive method to monitor BH_4_ and its oxidation products in biological/clinical samples is required for establishing its pharmacokinetic properties and to optimize the administration protocol.

## 5. Conclusions

Our study on standard stability suggests that DTE and DTPA are essential additives for the stabilization of BH_4_ under storage conditions, while for BH_2_ standards, the addition of HCl is sufficient (HPLC protocol A). With the modified HPLC/ECD method (protocol B) we were able to directly measure BH_4_ concentrations in the heart, kidney and brain of control and LPS-treated septic rats. Unfortunately, we were not able to detect BH_2_ concentrations in our tissue samples despite clear BH_2_ peaks in these preparations upon spiking the sample with authentic BH_2_ standard (not shown). We were also not able to establish a significant decrease in BH_4_ levels in the aorta of diabetic rats (after separation of BH_4_ from ascorbate) but observed a significant increase of BH_4_ concentrations in the heart and kidney and also a clear trend of increased BH_4_ levels in the brains of septic rats. The data presented herein provide information on BH_4_/BH_2_ storage conditions and the direct quantification of both biomolecules, especially BH_4_ by HPLC/ECD, for researchers interested in the measurement of BH_4_/BH_2_ in different tissues.

## Figures and Tables

**Table 1:** Experimental parameters for HPLC/ECD-based detection of BH4 and BH2 used in this study.

Parameters	Specification
HPLC system	Dionex UltiMate 3000 (Thermo Fisher Scientific)
Column	Synergi Polar, 250 mm × 4.6 mm, 4 µm, 80 Å (Phenomenex)
Column temperature	37 °C
Mobile phase	^a^ 50 mM potassium phosphate, pH 2.6, 0.1 mM DTE, 0.1 mM DTPA ^b^ 50 mM potassium phosphate, pH 4.5
Flow rate	^a^ 0.7 mL/min ^b^ 1.0 mL/min
Run time per sample	15 min
Injection volume	20 µL
Autosampler temperature	4 °C
Detector	Dionex CoulArray™(Thermo Fisher Scientific)
Autosampler temperature	4 °C
Detector settings	
- Electrode potentials (mV vs. palladium reference electrode)	^a^ 0; +150; +280; +365; +600 ^b^ 0; +150; +280
- BH_4_ quantification channel(s)	^a^ 150 mV + 280 mV ^b^ 0 mV
- BH_2_ quantification channel(s)	^a^ 600 mV ^b^ 280 mV
- Accuracy (deviation from the expected calibration curve value) ^b^	7.99% (0 mV, 1 µM BH_4_, *n* = 16) −3.11% (280 mV, 1 µM BH_2_, *n* = 15)
- Precision (deviation from the measured mean) ^b^	11.27% (0 mV, 1 µM BH_4_, *n* = 16) 3.36% (280 mV, 1 µM BH_2_, *n* = 15)
- Noise ^b^	3.13 ± 0.0.82 nA (0 mV, BH_4_, *n* = 10) 0.49 ± 0.0.16 nA (280 mV, BH_2_, *n* = 10)
- Limit of quantification, peak height and quantity of material (S/N = 10) ^b^	31.3 nA, 4.49 pmol (0 mV, BH_4_) 4.9 nA, 0.38 pmol (280 mV, BH_2_)
- Limit of detection, peak height and quantity of material (S/N = 3) ^b^	9.4 nA, 1.35 pmol (0 mV, BH_4_) 1.5 nA, 0.11 pmol (280 mV, BH_2_)

^a^ Protocol A; ^b^ Protocol B (optimized for separation of BH4 and ascorbate in biological samples).

**Table 2:** BH_4_ and BH_2_ (+biopterin [BP]) levels in different tissues in various animal disease models determined by different methods.

Species/Strain	BH_4_ [pmol/mg Protein]			BH_2_ [pmol/mg Protein]		Reference & Method
Wistar rats, healthy control and LPS-induced sepsis	Heart: Ctr ^1^: 0.3 LPS ^2^: 7.5	Kidney: Ctr: 0.5 LPS: 5.2	Brain: Ctr: 78 LPS: 133	n.d. ^3^		Present work HPLC/ECD
Healthy C57BL/CBA mice, GTPCH-I-deficient ^4^ Hph-1 ^5^ mice	Aorta: Ctr: 100–120 Hph-1: 50–60			n.d.		[33] HPLC/ECD
Healthy C3/HeN mice and septic LPS-treated mice	Brain: Ctr: 4.2 LPS: 7			n.d.		[34] HPLC/fluorescence with differential oxidation
Healthy C57BL/6 mice—young versus old	Aorta: Young: 8 Old: 5.8			Aorta: Young: 6 Old: 4		[35] HPLC/fluorescence with differential oxidation
C57BL/6 mice and ApoE^Tm1Unc^ mice with Western diet (HFD ^6^)	Aorta: Ctr + HFD: 7 ApoE + HFD: 7–37	Brain: Ctr + HFD: 20–25 ApoE + HFD: 25–30	Endothelial cells Ctr + HFD: 10 ApoE + HFD: 25	Aorta: Ctr + HFD: 5 ApoE + HFD: 5	Brain: Ctr + HFD: 1–2 ApoE + HFD: 1–2	[36] HPLC/fluorescence with differential oxidation
C57BL/6 mice and GTPCH-I overexpressing mice (tg-GCH ^7^)	Aorta: Ctr: 2.7 tg-GCH: 12			Aorta: Ctr: 3.7 tg-GCH: 16		[37] HPLC/fluorescence with differential oxidation
New Zealand white rabbits (healthy, hyperglycemic, treatments)	Heart: Ctr: 7.6; HG ^8^: 6; IPC ^9^: 10.2; HG + IPC: 7 Ctr + SEP ^10^: 11; HG + SEP: 6; IPC + SEP: 14; HG + IPC + SEP: 13 Ctr + DAHP ^11^: 6; IPC + DAHP: 10			n.d.		[28] HPLC/ECD
Rats, healthy (ZL ^12^) versus diabetic (ZDF ^13^)	Kidney: ZL: 6.5 (22 w) ZDF: 2.5 (22 w) ZDF + ebselen: 6.5 (22 w)			Kidney: ZL: <1 (22 w) ZDF: 5 (22 w) ZDF + ebselen: <1 (22 w)		[30] HPLC/ECD
Rats, healthy (ZL) versus diabetic (ZDF)	Lung: ZL: 2.2 (8 w); ZL: 1.9 (22 w) ZDF: 1.9 (8 w); ZDF: 0.8 (22 w) ZDF + ebselen: 1.3 (22 w)			Lung: ZL: 0.1 (8 w); ZL: 0.3 (22 w) ZDF: 0.2 (8 w); ZDF: 1.1 (22 w) ZDF + ebselen: 0.4 (22 w)		[38] HPLC/ECD
C57BL/6 mice and DOCA ^14^ salt hypertension, treatments	Aorta: Ctr: 110; Ctr + BH4: 130 DOCA: 50; DOCA (p47phox^−/−^): 90; DOCA (eNOS^−/−^): 70; DOCA + BH4: 90			n.d.		[9] HPLC/fluorescence with differential oxidation
ApoE^−/−^ mice with oral versus *i.v.* BH_4_ administration	Plasma: Peak value (*p.o.*): 423 nmol/L Peak value (*i.v.*): 2004 nmol/L			n.d.		[39] LC/MS ^15^
Healthy volunteers	Plasma: Age of 20: 19.5 nmol/L Age of 60: 6.6 nmol/L			n.d.		[40] LC/MS, derivatization with benzoyl chloride
Rats, healthy versus ischemia by ligation of the carotid artery	Brain: Sham: 4.5–7 Ischemia: 5–5.7			n.d.		[41] LC/MS
Diabetic patients with kidney disease	Plasma: Normoalbuminuria: 21.6 nmol/L Microalbuminuria: 12.9 nmol/L Microalbuminuria: 5.0 nmol/L			n.d.		[42] LC/MS

^1^ Ctr, control; ^2^ LPS, LPS induced sepsis; ^3^ n.d., not determined; ^4^ GTPCH-I, GTP-cyclohydrolase-I; ^5^ Hph-1, hyperphenylalaninemic mouse mutant 90% deficiency GTPCH-I; ^6^ HFD, high-fat (Western) diet; ^7^ tg-GCH, endothelial GTPCH transgenic; ^8^ HG, hyperglycemia; ^9^ IPC, ischemic preconditioning; ^10^ SEP, sepiapterin; ^11^ DAHP, diamino-6-hydroxypyrimidine, an inhibitor of BH_4_ synthesis; ^12^ ZL, Zucker nondiabetic lean rat; ^13^ ZDF, Zucker diabetic fatty rat; ^14^ DOCA, deoxycorticosterone acetate; ^15^ LC/MS, liquid chromatography coupled with mass spectrometry.

## Data Availability

Data are all contained within this article. Raw data are available from the corresponding author upon reasonable request.

## Abbreviations

BH2, 7,8-dihydrobiopterin; BH4, tetrahydrobiopterin; CoulArray, coulometric array system; DHFR, dihydrofolate reductase; DTE, 1,4-dithioerythritol; DTPA, diethylenetriaminepentaacetic acid; GTPCH-I, GTP cyclohydrolase I; HPLC/ECD, high performance liquid chromatography with electrochemical detection; LC-MS/MS, liquid chromatography–mass spectrometry; NOS, nitric oxide synthase; STZ, streptozotocin.
